# The cancer testis antigen TDRD1 regulates prostate cancer proliferation by associating with snRNP biogenesis machinery

**DOI:** 10.21203/rs.3.rs-2035901/v1

**Published:** 2023-02-22

**Authors:** Qin Feng, Hong Kim, Amrita Barua, Luping Huang, Modupeola Bolaji, Sharon Zachariah, Sung Yun Jung, Bin He, Tianyi Zhou, Aroshi Mitra

**Affiliations:** University of Houston; Baylor College of Medicine

## Abstract

Prostate cancer is the most commonly diagnosed noncutaneous cancer in American men. TDRD1, a germ cell-specific gene, is erroneously expressed in more than half of prostate tumors, but its role in prostate cancer development remains elusive. In this study, we identified a PRMT5-TDRD1 signaling axis that regulates the proliferation of prostate cancer cells. PRMT5 is a protein arginine methyltransferase essential for small nuclear ribonucleoprotein (snRNP) biogenesis. Methylation of Sm proteins by PRMT5 is a critical initiation step for assembling snRNPs in the cytoplasm, and the final snRNP assembly takes place in Cajal bodies in the nucleus. By mass spectrum analysis, we found that TDRD1 interacts with multiple subunits of the snRNP biogenesis machinery. In the cytoplasm, TDRD1 interacts with methylated Sm proteins in a PRMT5-dependent manner. In the nucleus, TDRD1 interacts with Coilin, the scaffold protein of Cajal bodies. Ablation of TDRD1 in prostate cancer cells disrupted the integrity of Cajal bodies, affected the snRNP biogenesis, and reduced cell proliferation. Taken together, this study represents the first characterization of TDRD1 functions in prostate cancer development and suggests TDRD1 as a potential therapeutic target for prostate cancer treatment.

## Introduction

*TDRD1 (Tudor Domain Containing 1)* is a germ cell-specific gene solely expressed in human testes and ovaries under physiological conditions but not in any other normal tissues. However, in up to 68% of prostate tumors, TDRD1 is erroneously overexpressed, and its expression levels strongly correlate with TMPRSS2-ERG gene fusion. Indeed, we and others confirmed that *TDRD1* is a *bona fide* ERG target gene[1–3]. While TDRD1 overexpression is present in nearly all ERG-expressing primary tumors, some tumors express TDRD1 even without TMPRSS2-ERG fusion[1].

As its name indicates, TDRD1 contains 4 Tudor domains, which are conserved protein structural domains with approximately 60 amino acids in length. Tudor domains have been identified as epigenetic ‘readers’ that bind to methylated lysine and arginine residues through their aromatic-binding cage structure[4]. Being a germ cell-specific protein, TDRD1 acts as a scaffold protein and interacts with several piRNA processing proteins through its Tudor domains in mouse testis. A complete *Tdrd1* knockout in mouse abolished the piRNA biogenesis pathway and led to male infertility[5, 6].

On the other hand, it has become increasingly recognized that the protein arginine methyltransferase (PRMT) family of enzymes is involved in cancer development[7]. Two types of PRMT proteins catalyze dimethylation on arginine residues. Type I PRMTs produce asymmetric dimethylarginine (aDMA); whereas the type II PRMTs produce symmetric dimethylarginine (sDMA). PRMT5 is the major type II PRMT and is overexpressed in many types of cancers, including leukemia/lymphoma, glioblastoma, melanoma, as well as prostate cancer[8–10]. Interestingly, it was reported that PRMT5 protein has opposite roles on prostate cancer cell growth depending on its subcellular localization. The nuclear PRMT5 protein inhibits prostate tumor growth, whereas cytoplasmic PRMT5 promotes tumor growth[11]. Consistent with this finding, in prostate premalignant and cancer tissues, PRMT5 mainly accumulates in the cytoplasm, and its expression and methyltransferase activity is essential for cancer cells to grow[11]. These studies imply the importance of cytoplasmic substrates of PRMT5 in prostate cancer cell growth. However, in contrast to well-documented evidence on nuclear substrates of PRMT5 and their direct roles in transcriptional regulation, little is known about the cytoplasmic function of PRMT5 in prostate cancer.

Studies have shown that PRMT5 methylates Sm (smith core) proteins, and this event is an essential initiation step for assembly of small nuclear ribonucleoproteins (snRNPs) in the cytoplasm. Partially assembled snRNP is transported to the nucleus and further matured in non-membrane bound nuclear bodies named Cajal bodies. There are very few studies on the role of Sm proteins and snRNP biogenesis in prostate cancer, but two interesting reports showed that SNRPE, also known as SmE, is overexpressed in high-grade prostate cancer cells[12, 13]. Knockdown of SNRPE suppressed prostate cancer cell proliferation, while overexpression of SNRPE promoted cancer cell proliferation[12]. These findings suggest that PRMT5-mediated Sm protein methylation and snRNP assembly likely play an important role in sustaining the growth of prostate cancer cells.

In this study, we found that in prostate cancer cells, TDRD1 is associated with important proteins in snRNP assembly in both the cytoplasm and the nucleus. Cytoplasmic TDRD1 interacts with methylated Sm proteins in a PRMT5-dependent manner, and nuclear TDRD1 interacts with Coilin, the scaffold protein of Cajal bodies. Ablation of TDRD1 in prostate cancer cells by CRISPR-Cas9 disrupted the cellular localization of Coilin and the production of snRNAs. TDRD1 perturbation activated the tumor suppressor p53 and significantly impaired prostate cancer cell proliferation. In addition, depletion of TDRD1 in VCaP cells increased sensitivity to antiandrogens, while overexpression in 22Rv1 cells enhanced resistance. Our study reveals a novel function of TDRD1 and suggests TDRD1 as a potential therapeutic target for prostate cancer treatment.

## Materials And Methods

### Cell culture, transient transfection, and siRNA knockdown

VCaP, HeLa, and 293T cells were grown in DMEM supplemented with 10% FBS and 1% penicillin/streptomycin. 22Rv1 and LNCaP cells were grown in RPMI1640 supplemented with 10% FBS and 1% penicillin/streptomycin. Mycoplasma contamination testing has been performed every six months using Universal Mycoplasma Detection Kit (ATCC #30-1012K). Transient transfection was performed using TransIT LT1 (Mirus Bio, WI, USA) for HeLa and 293T cells. PEI-Max was used for transient transfection of 22Rv1 and LNCaP cells (Polysciences, PA, USA). ON-TARGETplus SMARTpool siRNA was used for siRNA knockdown.

### CRISPR/Cas9-mediated TDRD1 and ERG knockout

To generate TDRD1 knockout VCaP cell lines, two different types of TDRD1 sgRNA oligonucleotides were used: Lentivirus-based hPGK-puro-2A-tBFP vector (sgRNA sequence: 5’-GAT ATG GCT TGA AAC CCA GTG G-3’) from Sigma-Aldrich (MO, USA) and edit-R lentiviral vector (sgRNA sequence: 3’-ACA TGC TGT GGA GCA ATA GT-5’) and the non-targeting control vector(U-009501-01-02) were obtained from Dharmacon (CO, USA). The ERG lentiviral sgRNA vector was purchased from Sigma-Aldrich with the sgRNA sequence: 5’-CCG TGG AGA GTT TTG TAA GGC T-3’. VCaP cells were transduced with lentivirus expressing Cas9 and then selected with 5 μg/ml blasticidin (Sigma-Aldrich #15205), followed by transduction with lentiviruses containing TDRD1 and ERG sgRNAs and control, followed by a selection of 5 μg/ml puromycin (Sigma-Aldrich #P8833).

### Cell growth assay

VCaP, 22Rv1, and LNCaP cells were seeded at a density of 1 × 10^4^ cells per well in flat-bottomed 96-well plates and grew for 3–11 days. CellTiter-Glo^®^ Luminescent Cell Viability Assay (Promega, Madison, WI, USA) was used to measure cell viability every other day, and the luminescence was determined by Synergy^™^ neo2 multi-mode reader (BioTek, Winooski, VT, USA).

### Cloning and plasmids

The pEGFP-C3 vector (Clonetech/Takara Bio, San Jose, CA, USA) was used to clone all GFP-fused TDRD1 fragments and mutants. The amino acid positions of each clone are: MYND, a.a.1-253; eTD1, a.a. 233–493; eTD2, a.a.481–715; eTD3, a.a.692–939; eTD4, a.a.911–1180. TDRD1 and Coilin deletion mutants were generated using the Q5 site-directed mutagenesis kit (NEB, Ipswich, MA, USA, # E0554S) following the manufacturer’s instruction. The wild-type and R5K SNRPD3 expressing vectors were generated by DNA assembly using NEBuilder HiFi DNA Assembly kit (NEB #E2621L). pEGFP-hCoilin plasmid was obtained from Addgene (#36906) and further subcloned to pSG5-based expression vector. The recombinant TDRD1 eTD4 was cloned into pET45b(+) vector (Novagen #71327, Reno, Nevada, USA).

### Western blot, Co-immunoprecipitation, and antibodies

Cells were washed with ice-cold PBS and lysed with lysis buffer (50 mM Tris-HCl, pH 7.5, 100 mM NaCl, 0.1% NP-40, 50 mM NaF, 1 mM DTT, 1 mM PMSF, and 1X Protease inhibitor cocktail). After centrifugation, the supernatant was collected for immunoprecipitation or Western blot analysis. For the Co-IP experiment, 2 mg antibody and 25 μl protein G beads were used for incubation with lysate at 4°C for 4 hrs. After 3 extensive washes with lysis buffer, the immunoprecipitated proteins were analyzed by western blot. Antibodies used include: α-β-Actin (Santa Cruz Biotechnology, Dallas, TX, USA, #sc-47778), α-GFP (Santa Cruz Biotechnology #sc-9996), α-SNRPD3 (Sigma-Aldrich #HPA001170), α-Flag-tagged HRP (Sigma-Aldrich #A8592), α-Coilin (Cell Signaling, Danvers, MA, USA, #14168), α-PARP1 (Santa Cruz Biotechnology #sd-25780), α-p53 (Santa Cruz Biotechnology #sc-126), α-phospho-p53 (pSer15) (Santa Cruz Biotechnology #sc-101762), α-p21 (Waf/Cip1, Cell Signaling #2947). The antibodies used in Co-IP were α-GFP (ProteinTech, Rosemont, IL, USA, #50430-2-AP), α-Flag (Sigma-Aldrich #M8823), α-Coilin (Abcam, Cambridge, UK, #ab87913), α-ERG (Abcam #ab92513), and α-IgG (Santa Cruz Biotechnology #sc-2025). The TDRD1 monoclonal antibody was generated in-house and published previously[1].

### Recombinant protein purification

The recombinant TDRD1 eTD4 was expressed in BL21(DE3) *E. Coli Strain* (NEB #C2527H). Expression of eTD4 was induced with 0.4 mM IPTG at 30°C for 5 hours and purified using Ni-NTA agarose (Qiagen #30210, MA, USA) following the manufacturer’s protocol.

### Peptide pull-down assay

Peptides containing the C-terminal sequence of SNRPD3 and SNRPD1 were synthesized by GenScript (Piscataway, NJ, USA). These peptides were all biotinylated at the N-termini. After transient transfection, 293T cells were harvested and the cell lysate was used for peptide pull-down. M280-streptavidin Dynabeads (Invitrogen #11205D) and 2 mg of each biotinylated peptide were added to the lysate. After incubation at 4°C with rotation for 4 hrs, beads were subjected to extensive wash, and the precipitated proteins were separated by SDS-PAGE and analyzed by western blot.

### Mass spectrum assay

Following the immunoprecipitation, Ni-NTA beads were washed and then boiled in 30 ul of 1× NUPAGE^®^ LDS sample buffer and subjected to SDS-PAGE. After being visualized by Coomassie blue stain, the gel was excised, destained and subjected to in-gel digestion using 100 ng of trypsin (GenDepot T9600, Barker, TX, USA). The tryptic peptides were extracted in 0.1% formic acid and applied to nanoHPLC–MS/MS system, which consists of a nano-LC 1000 system (Thermo Scientific) and a Q Exactive^™^ Plus (Thermo Scientific) mass spectrometer. The peptides were loaded onto a Reprosil-Pur Basic C18 pre-column (size: 2 cm × 100 μm). The pre-column was switched in-line with an analytical column (size: 50 mm × 150 um) that was packed with Reprosil-Pur Basic C18 equilibrated in 0.1% formic acid. The peptides were eluted through a 75-min discontinuous gradient of 4–26% acetonitrile in 0.1% formic acid. The peptides were then electro-sprayed into mass spectrometer that was operated in the data-dependent acquisition mode acquiring fragmentation spectra of the top 50 strongest ions. Obtained MS/MS spectra were parsed against a target-decoy human refseq database in Proteome Discoverer 1.4 (Thermo Fisher) with the Mascot algorithm (Mascot 2.4, Matrix Science). The precursor mass tolerance was confined within 20 ppm with a fragment mass tolerance of 0.5 Da and a maximum of four missed cleavage allowed. The peptides identified from the Mascot result file were validated with 5% false discovery rate (FDR). For relative quantification, the data was then grouped into gene products and assigned homology and identification quality groups using an in-house developed algorithm. Each gene product amount was estimated using a label-free intensity-based absolute quantification (iBAQ) approach as previously reported[14].

### Cell cycle analysis

Cell cycle analysis was performed by Propidium Iodide (PI) staining (Sigma-Aldrich #P4864) and FACS. Briefly, cells were harvested and washed three times with ice-cold PBS. Cells were then fixed with 70% ethanol drop-by-drop when being vortexed softly. Cell were then treated with 0.25% Triton-X-100 (Sigma-Aldrich #T9284) to break the membrane. After two washes with ice-cold PBS, cells were incubated with 100 ug RNase A (Sigma-Aldrich #R6513) for 30 mins at 37 °C. After washing, cells were stained with PI at a concentration of 25 mg/ml for overnight at 4°C before FACS analysis (BD Fortessa X20, Franklin Lakes, NJ, USA). The cell cycle patterns were analyzed by FlowJo software (v10.6.1_CL).

### Immunofluorescence

The cells were seeded in each well of 6-well plates with a sterilized 1.5 mm thickness cover-glass coated by 0.01% Poly-L-lysine (Sigma-Aldrich #P8920). After incubation of cells for 24 h, the cells were fixed and permeabilized with ice-cold 100% methanol for 15 mins at −20°C. After blocking, cells were incubated with primary antibodies for 2 h and secondary antibody Alexa Fluor 594 for another 2 h at room temperature. After mounting, confocal images were acquired using Olympus FV3000 at the Biology & Biochemistry Imaging Core (BBIC) at the University of Houston. The Corrected Total Cell Fluorescence (CTCF) = Integrated Density – (Area of selected cell X Mean fluorescence of background readings) was quantified following the instruction of measuring cell fluorescence method of Image J software (NIH, Bethesda, MD, USA).

### TCGA data analysis

The TCGA_PRAD and WCDT_MCRPC datasets from TCGA were downloaded using the GDCquery function from TCGAbiolinks[15]. The data processing and preparation of the expression matrix were done using GDCprepare from TCGAbiolinks. The matrix was then normalized using TCGA_normalize and visualized in Prism[16].

### Xenograft tumor growth and immunohistochemistry (IHC) staining

NSG (NOD.Cg-*Prkdc^scid^* *Il2rg^tm1Wjl^*/SzJ) mice from Jackson Laboratory (Bar Harbor, ME, USA) were used for subcutaneous xenografts. Control or TDRD1 KO VCaP cells were resuspended with100μl of 1×PBS and Matrigel and injected into the flank area of randomized male NSG mice 4–5 months of age. Tumor growth was measured weekly after injection by using a caliper. Tumor volume was calculated according to the following formula: 4/3n*(Length/2)*(Width/2)^2^. After tumor tissues were harvested and weighted, they were fixed in 10% buffered formalin, embedded in paraffin, sectioned, and stained. We used the VECTASTAIN Elite ABC-HRP reagent, Peroxidase, R.T.U. kit (#PK-7100) for IHC staining following the manufacturer’s instructions. The Ki67 antibody was obtained from Thermo Scientific (#RM-9106-S0) and diluted 1:200 for overnight incubation at 4°C. The TDRD1 monoclonal antibody was generated at the Protein and Monoclonal Antibody Production Core at Baylor College of Medicine (Houston, TX, USA) [1]. The secondary antibody was biotinylated horse anti-rabbit IgG, R.T.U (#BP-1100-50) or horse anti-mouse IgG, R.T.U (#BP-2000-50) from Vector laboratories (Newark, CA, USA). Cytation 5 and Gen5 Image + software were used to quantify the IHC signal intensity on the prostate cancer tissue microarray, which was commercially available from Novus Biologicals (NBP2-30169) (Centennial, CO, USA).

### Statistics

Data in this study were analyzed using Prizm 8.0 (GraphPad, San Diego, CA, USA). The sample size was set to a minimum of three independent experiments (biological repeats) and experimental findings were reliably reproducible. Statistical significance of between-group differences was determined by non-paired Student’s *t*-test. The N numbers of biological replicates were indicated in the figure legends. Differences were considered statistically significant at p ≤ 0.05. The pair-wise gene expression correlation analysis done in GEPIA uses methods including Pearson, Spearman and Kendall (http://gepia.cancer-pku.cn).

### Study approval

The mouse experiments were performed under the protocol (AUP-0121-0002) approved by IACUC at the Houston Methodist Research Institute and protocol (PROTO202000026) approved by IACUC at the University of Houston.

## Results

### TDRD1 is important for cell proliferation in TDRD1-positive prostate cancer cell line

**Figure 1:**
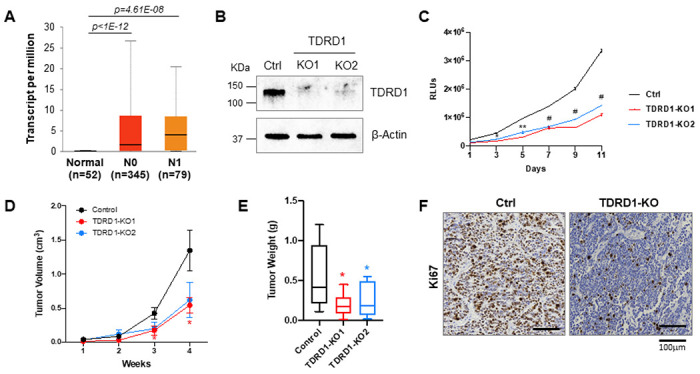
TDRD1 ablation in VCaP cells reduced cell proliferation. ***(A)*** Expression of TDRD1 in prostate adenocarcinoma based on nodal metastasis status in TCGA samples. The figure was generated from the UALCAN cancer database. N0, no regional lymph node metastasis. N1, metastases in 1 to 3 axillary lymph nodes. ***(B)*** TDRD1 was knocked out in VCaP cells by CRISPR/Cas9 genome editing. Total cell lysates from one control and two KO cells with different gRNAs were subjected to Western blot analysis. Ctrl: mock knockout with a non-targeting gRNA. ***(C)*** Knockout of TDRD1 reduced the growth of VCaP cells. N=4. Error bars, SEM. *, p<0.05. **, p<0.01. #, p<0.001 by Student *t*-test. The statistical labels apply to both TDRD1 KO lines compared to the control cells. ***(D)*** Control mock knockout and two TDRD1 KO VCaP cells were injected subcutaneously into NSG xenograft mice. Tumor volumes were determined. Error bars, SEM. *, p<0.05. The statistical labels apply to both TDRD1 KO tumors compared to the control tumors. ***(E)*** Weight of the tumors collected in (D). Box and whiskers show Min to Max. N: Control, 9. TDRD1-KO1, 10. TDRD1-KO2, 12. *, p<0.05 by Student *t*-test. ***(F)*** A representative Ki67 immunohistochemical staining of tumor tissues obtained in (D). The nuclei were counterstained with hematoxylin. *, p<0.05. **, p<0.01 by Student *t*-test.

TDRD1 gene is known to be overexpressed in primary prostate tumors[1, 2]. Cancer OMICS data from TCGA further showed that TDRD1 overexpression is preserved in prostate tumors regardless of nodal metastasis status, indicating that TDRD1 is likely indispensable in established prostate cancer cells (Fig. 1A). To investigate the biological function of TDRD1 in these cancer cells, we tried to deplete TDRD1 in TDRD1-positive VCaP cells using RNA-guided CRISPR-Cas9 system. We attempted but were not able to obtain single colonies of cells with a successful knockout of TDRD1, suggesting that TDRD1 might be essential for VCaP cell survival. Eventually, we obtained two pooled TDRD1 knockout cell populations from two different sgRNAs. Both pools showed high knockout efficiency (Fig. 1B).

As expected, both TDRD1-KO1 and KO2 cells showed significantly reduced growth rates than control VCaP cells (Fig. 1C), indicating that TDRD1 is important for VCaP cell proliferation. We further inoculated TDRD1-KO1 cells to the immunodeficient male NSG mice subcutaneously and monitored the tumor growth. As shown in Fig. 1D and 1E, a similar growth inhibitory effect was observed when TDRD1 knockout VCaP cells grew *in vivo*. Furthermore, we collected these tumors and performed immunohistochemical staining to examine the level of Ki67 in these tumors. As shown in Fig. 1F, we observed overall more Ki-67-positive cells in TDRD1-WT tumors than in TDRD1-KO tumors, confirming that ablation of TDRD1 reduces the VCaP cell proliferation both *in vitro* and *in vivo* as xenografted tumors in mice.

### TDRD1 is present in both the cytoplasm and nucleus of prostate cancer cells

**Figure 2:**
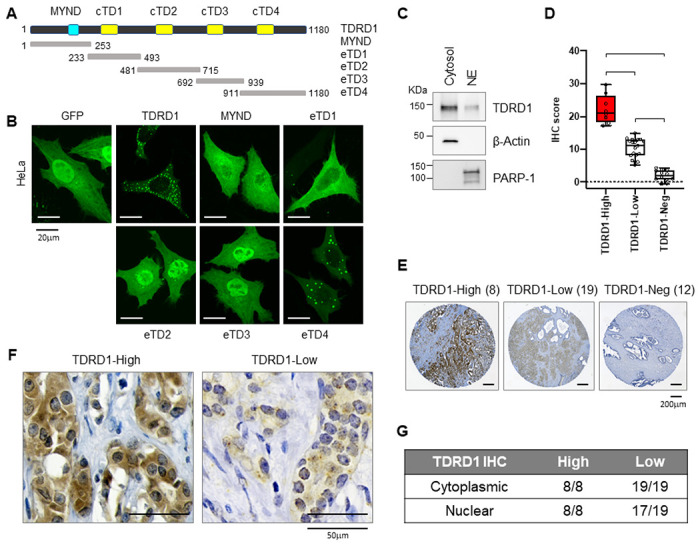
TDRD1 is present in both cytoplasm and nucleus, but primarily in the cytoplasm. ***(A)*** Schematic diagram of TDRD1 functional domains. Amino acid positions of each domain are: MYND, 170-205; Tudor 1, 312-372; Tudor 2, 541-600; Tudor 3, 762-821; Tudor 4, 990-1048. cTDs, core Tudor domains. eTDs, extended Tudor domains. ***(B)*** immunofluorescent staining of TDRD1 deletion mutants in the form of GFP-fusion in HeLa cells. ***(C)*** The cellular distribution of TDRD1 in VCaP cells was determined by Western blot analysis after cell fractionation into the cytosol and nuclear extract (NE). ***(D)*** The 39 prostate tumors were grouped into TDRD1-high, -low, and -negative based on the TDRD1 IHC staining intensity. ***, p<0.001 by Student *t*-test. ***(E)*** The representative TDRD1 IHC in prostate tumors from each group. ***(F)*** DRD1 is found in both cytoplasm and nuclei of tumor cells, with a predominance in the cytoplasm. ***(G)*** The frequency of tumors displaying positive TDRD1 staining in the cytoplasm and nucleus.

Previously published studies of TDRD1 have established its critical role in the regulation of piRNA biogenesis in germ cells[17–19]. However, in mammals, piRNA is only found in testes and ovaries[17, 20], suggesting that erroneously expressed TDRD1 must have a piRNA-independent role in prostate cancer cells. To investigate how TDRD1 regulates cell proliferation in prostate cancer cells, we sought to identify TDRD1-interacting proteins based on TDRD1 functional domains. Human TDRD1 protein contains five functional domains, including a MYND-type of domain (amino acids 170–206), and four Tudor domains scattered on TDRD1 protein (Fig. 2A). TDRD1 belongs to the Tudor domain-containing protein (TDRD) family, which all contain one or more extended Tudor domains. Each extended Tudor domain contains a core Tudor domain (cTD) that is highly homologous to the prototype Tudor domain identified in SMN1 (Survival of Motor Neuron 1)[21]. We first made a series of TDRD1 deletion mutants and fused them with the green fluorescent protein (GFP). These deletion mutants are named eTDs because they contain respective extended Tudor domains (Fig. 2A). When transiently expressed in HeLa cells, the full-length TDRD1 proteins formed speckles in both the cytoplasm and the nuclei, but mainly localized in the cytoplasm. eTD4 exhibited a similar pattern as the full-length TDRD1 protein (Fig. 2B), indicating that eTD4 is responsible for the accurate subcellular localization of the full-length TDRD1 protein. The cellular localization of endogenous TDRD1 was further confirmed by cell fractionation in VCaP cells (Fig. 2C). Moreover, we examined the localization of TDRD1 in prostate tumor samples from a commercially available tissue microarray, which contains 39 human prostate tumor biopsy samples. We performed TDRD1 IHC staining as previously described [1]. The TDRD1 antibody successfully differentiated between the wild-type VCaP xenograft tumor and the TDRD1-KO tumor in IHC staining. (**Fig. S1A**). Based on the TDRD1 IHC score quantified by the Cytation 5 Image + software, we were able to classify the tumor samples into three groups, with 8 TDRD1-High tumors, 19 TDRD1-Low tumors, and 12 TDRD1-Negative tumors (Figs. 2D and 2E). In most TDRD1-High and -Low tumors, we observed positive TDRD1 staining in both cytosol and nuclei (Fig. 2F). **Figs. S1B and S1C** illustrate the quantification of TDRD1 staining in the cytoplasm and nucleus of each TDRD1-positive tumor. The frequency of tumors displaying positive TDRD1 staining is summarized in Fig. 2G. Taken together, these results show that TDRD1 is present in both the cytoplasm and nucleus of prostate cancer cells, with a predominant presence in the cytoplasm.

### Cytoplasmic TDRD1 interacts with Sm proteins in a methylation-dependent manner

**Figure 3:**
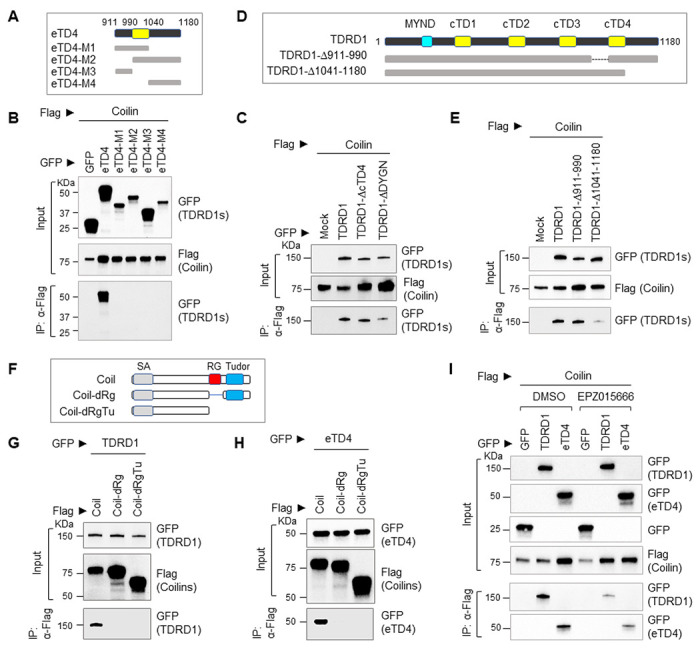
Cytoplasmic TDRD1 protein interacts with Sm proteins. ***(A)*** 152 TDRD1-interacting proteins were identified from the cytoplasmic fraction of VCaP cells by Mass spectrum analysis (fold enrichment >2). KEGG pathway analysis was performed by DAVID Bioinformatics Resources, and the pathways that show P value<0.01 are listed. ***(B)*** snRNP proteins are enriched in eTD4-interacting proteins. The abundance of proteins identified by the Mass spectrum is shown as iBAQ. The enrichment of each protein has been calculated by the percentage of increased iBAQ in eTD4 pull-down. All snRNP proteins are marked in red. ***(C)*** Peptide pull-down assay using synthesized biotinylated peptides. The input samples are total 293T cell lysate exogenously expressing GFP-fused TDRD1 deletion mutants. sm, symmetrically di-methylated. am, asymmetrically di-methylated. ***(D)*** Co-IP experiment to validate the interaction between SNRPD3 and TDRD1. GFP-tagged TDRD1 and eTD4 were exogenously expressed in 293T cells. Anti-GFP antibody was used to co-immunoprecipitate endogenous SNRPD3. EPZ015666: a selective RPMT5 inhibitor. ***(E)*** Co-IP experiment to validate the interaction between endogenous SNRPD3 and TDRD1 in VCaP cells. Input: 5% of VCaP cell lysate used for each Co-IP. VCaP cells were treated with 5 mM of EPZ015666 for 24 hours before harvest.

To further understand the function of TDRD1, we decided to generate the smaller eTD4 recombinant protein and to identify its cytoplasmic interacting proteins. We purified 6His-tagged eTD4 protein and used it as bait in a pull-down assay followed by mass spectrometry analysis. BSA protein was used as a control for the bait. Using VCaP cell cytoplasmic fraction as an input, we identified 152 potential eTD4-specific interacting proteins in total. A full list of these 152 proteins is provided in **Supplemental Table 1.** KEGG pathway analysis indicated that the Spliceosome pathway is the most significantly enriched pathway in eTD4 interactome (Fig. 3A). Interestingly, nearly all the identified proteins enriched in this pathway are involved in snRNP biogenesis in the cytoplasm, and most of them are the Sm proteins (Fig. 3B), suggesting that TDRD1 may interact with these proteins through its eTD4 region. Among them, SNRPD1, SNRPD3, and SNRPB are known methylated proteins[22, 23]. Their C-terminal sequences contain multiple arginine residues that are subject to symmetrical dimethylation by PRMT5[24, 25]. Because Tudor domains exert their functions by recognizing and binding methylated arginine and lysine residues, we further validated the mass spectrometry result by a peptide pull-down experiment. We synthesized three peptides based on the C-terminal 32 residues of SNRPD3, which was the most abundant eTD4-interacting Sm protein in our mass spectrometry analysis (Fig. 3B). The sequence harbors 4 ‘RG’ sites that were documented as PRMT5 methylation sites previously[26]. The peptides are either unmodified, or symmetrically dimethylated (sDMA), or asymmetrically dimethylated (aDMA). We tested the binding between these peptides with all five functional domains of TDRD1. The result showed that the full-length TDRD1 and eTD4 selectively bound to the symmetrically dimethylated peptide, but not the unmodified or asymmetrically methylated peptides (Fig. 3C). Moreover, the interaction is specific to eTD4, as none of the other deletion mutants had this binding activity. Similar results were obtained when peptides composing C-terminal 29 residues of SNRPD1 were tested (**Fig. S2**). To further validate the interaction between TDRD1 and endogenous SNRPD3, co-immunoprecipitation (Co-IP) experiment was performed in cells treated with vehicle control or EPZ015666, a selective PRMT5 inhibitor. EPZ015666 significantly reduced the interaction between SNRPD3 and exogenously expressed TDRD1 or eTD4 in 293T cells (Fig. 3D). Similarly, the loss of interaction between endogenous SNRPD3 and TDRD1 was also observed in VCaP cells (Fig. 3E). These results further confirm that the interaction between TDRD1 and SNRPD3 is likely dependent on PRMT5 activity and mediated by symmetrically dimethylated arginine.

### TDRD1 interacts with SNRPD3 through its core Tudor 4 domain

**Figure 4:**
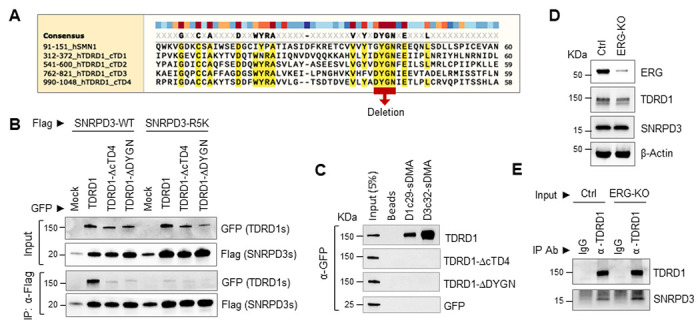
TDRD1 interacts with SNRPD3 through the cTD4 in a methylated arginine-dependent manner. ***(A)*** multiple alignments of SMN1 Tudor domain and 4 core Tudor domains of TDRD1. The 4 conserved amino acids, DYGN, have been deleted in TDRD1-ΔDYGN to disable methylated arginine-dependent binding in cTD4 . ***(B)***Co-IP experiment performed in HeLa cells. TDRD1 expression vectors are GFP-tagged, and SNRPD expression vectors are Flag-tagged. TDRD1-ΔcTD4 and TDRD1-ΔDYGN were constructed on the full-length TDRD1. R5K: 5 arginines have been mutated to lysines in the C-terminus of SNRPD3. Mock: empty vector control. ***(C)*** Peptide pull-down assay. 293T cell lysate exogenously expressing GFP-fused full-length TDRD1 or deletion mutants were used as input samples. ***(D)*** Western blot analysis of ERG, TDRD1, SNRPD3 in ERG-KO VCaP cells.***(E)*** Co-IP experiment to determine the interaction between TDRD1 and SNRPD3 in ERG-KO VCaP cells.

Since the eTD4 mutant contains a core Tudor 4 (cTD4) domain and long flanking sequences on both sides, we further made two additional mutants to examine if the cTD4 domain is required for the interaction. Figure 4A is an alignment of all four core Tudor domains with the prototypic Tudor domain of SMN1. We deleted the entire 61 amino acids of the cTD4 domain, or only 4 conserved residues ‘DYGN’ of cTD4 in the context of full-length TDRD1. Alternatively, we made arginine to lysine mutations on 5 ‘RG’ sites at the C-terminus of SNRPD3. These sites include 4 reported methylated ‘RG’ sites and 1 potential methylation site. The Co-IP experiment in Fig. 4B showed that disruption of either the TDRD1 cTD4 domain or SNRPD3 methylation sites abolished the interaction between TDRD1 and SNRPD3. We further examined the binding of symmetrically dimethylated peptides with cTD4 mutants. As shown in Fig. 4C, none of the methylated peptides retained the interaction with TDRD1 when cTD4 was disrupted. Collectively, these results demonstrated that TDRD1 specifically interacts with cytoplasmic PRMT5-methylated SNRPD proteins through its cTD4 domain.

TDRD1 was reported as an ERG target gene[1, 27]. We next sought to determine if ERG would affect the interaction between TDRD1 and SNRPD3. We generated ERG-KO VCaP cells using CRISPR-Cas9 gene editing. Although the protein level of TDRD1 has been slightly reduced in ERG-KO cells (Fig. 4D), the Co-IP result shown in Fig. 4E demonstrates that ERG gene deletion does not affect the interaction between TDRD1 and SNRPD3 in VCaP cells.

### Nuclear TDRD1 associates with Coilin

**Figure 5:**
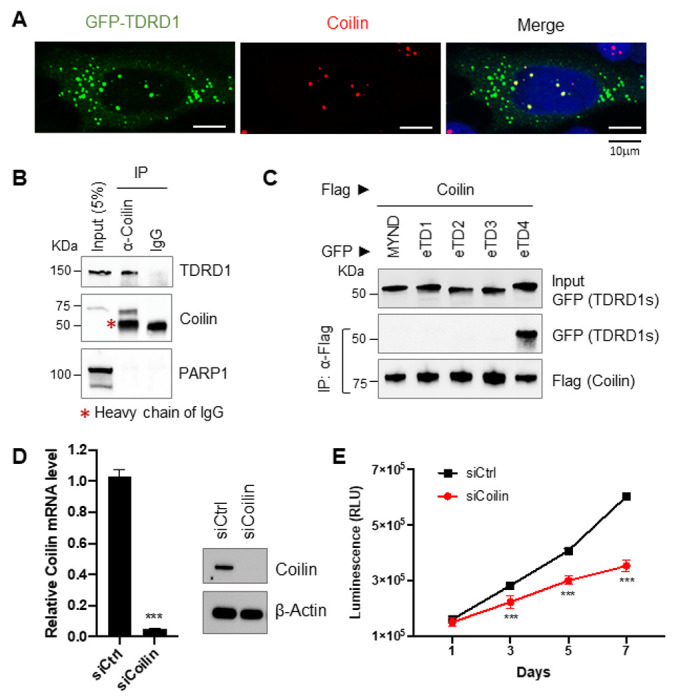
TDRD1 associates with Coilin in nuclei. ***(A)***Co-localization of TDRD1 (GFP) and Coilin (endogenous) in HeLa nuclei. ***(B)***Endogenous Coilin co-immunoprecipitated with endogenous TDRD1 in VCaP nuclear extract. ***(C)*** Deletion mapping identifies eTD4 as the Coilin-interaction regions on TDRD1 in HeLa cells. TDRD1 fragments were tagged with GFP and Coilin was Flag-tagged. (***D***) Knockdown of Coilin by siRNA in VCaP cells. RT-qPCR and Western blot analysis were performed to determine the mRNA level and protein level of Coilin. RNA samples were duplicated. ***, p<0.001 by *t*-test. (***E***) Coilin knockdown by siRNA reduced VCaP cell growth. N=3. ***, p<0.001 by *t*-test.

The interaction between TDRD1 and methylated Sm protein suggests that TDRD1 is likely implicated in the snRNP assembly. While the core snRNPs are assembled in the cytoplasm, the final snRNP assembly step takes place in the non-membrane structure Cajal bodies in the nucleus[28]. Because the protein Coilin is a marker of Cajal bodies and a small amount of TDRD1 was observed in the nucleus with a staining pattern similar to Coilin, we reasoned if TDRD1 may colocalize with Coilin (Fig. 2B). Indeed, by immunofluorescent staining, TDRD1 and Coilin exhibited a spatial colocalization in the nucleus (Fig. 5A). To further assess the interaction between endogenous TDRD1 and Coilin, we used the Coilin antibody to immunoprecipitate Coilin from VCaP cell nuclear extract, and TDRD1 was indeed co-immunoprecipitated with Coilin, but not with IgG. As a control, the nuclear marker protein PARP1 did not co-IP with Coilin despite its abundant expression in VCaP nuclear extract (Fig. 5B). By deletion mapping, we then narrowed down the Coilin-interacting region of TDRD1 to eTD4, which is the same region that interacts with methylated SNRPD3 (Fig. 5C). This interaction between nuclear TDRD1 and Coilin strongly suggests that Coilin might be also involved in regulation of VCaP cell proliferation. We chose to knock down Coilin by siRNA in VCaP cells and determined cell growth. As shown in Fig. 5D and 5E, Knockdown of Coilin significantly reduced the growth of VCaP cells, which is consistent with what we have observed from TDRD1 ablation.

### TDRD1 C-terminal sequence is essential for its interaction with Coilin

**Figure 6:**
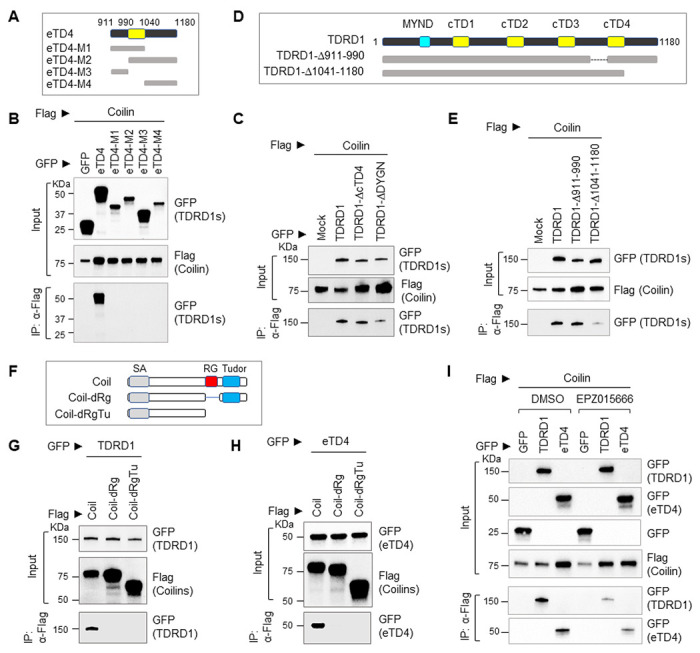
Mapping the interacting regions between TDRD1 and Coilin. ***(A)*** A schematic diagram of TDRD1 eTD4 deletion mutants used in (B). ***(B)*** Deletion mapping to define the Coilin-interacting regions on TDRD1 eTD4 by Co-IP. eTD4 mutants were GFP-tagged, and Coilin was Flag-tagged. ***(C)*** Co-IP experiment to determine if core Tudor 4 domain is necessary for Coilin interaction. ***(D and E)*** A schematic diagram of TDRD1 deletion mutants and their interactions with Coilin and SNRPD3 by Co-IP experiment. ***(F)***Deletion mutants made based on the functional domains of human Coilin. SA, Self-association domain. RG, Arginine-Glycine-rich box. ***(G)*** Co-IP experiment to determine the interaction between full-length TDRD1 and Coilin deletion mutants listed in (F). TDRD1 is GFP-tagged and Coilin mutants are Flag-tagged. ***(H)*** Co-IP experiment to determine the interaction between TDRD1 eTD4 and Coilin deletion mutants. ***(I)*** Co-IP experiment to determine if the interaction between TDRD1 and Coilin is dependent on the enzymatic activity of PRMT5. The transfected 293T cells were treated with DMSO or PRMT5-selective inhibitor EPZ015666 (5 uM) for 24 hours before cell harvest.

Next, we performed additional deletion mapping on eTD4 to precisely delineate the Coilin-binding region on TDRD1 (Fig. 6A). Interestingly, none of the deletion mutants preserved the binding activity of wildtype eTD4 (Fig. 6B). Furthermore, deletion of the cTD4 in the context of full-length TDRD1 did not affect the interaction, suggesting that the Coilin-interacting region does not overlap with the SNRPD3-interacting region on TDRD1, and the flanking regions of cTD4 may mediate the interaction with Coilin (Fig. 6C).

Because the structure of human eTD4 has not yet been reported, to gain more information on how eTD4 interacts with Coilin, we took advantage of newly developed AlphaFold, an AI system that predicts a protein’s three-dimensional structure based on its amino acid sequences[29]. There is very high confidence in the predicted structure of Tudor domains of the human TDRD1 structure (**Fig. S3A**). Within eTD4, the per-residue confidence scores (pLDDT) of amino acid residues between Q930 and F1118 are consistently higher than 70, and most importantly, the two tandemly arranged anti-parallel beta-sheet structures receive the highest pLDDT, indicating that the AlphaFold’s prediction of TDRD1 protein structure is highly accurate (**Fig. S3B**). While the cTD4 resembles the prototypical Tudor domain that was originally identified in SMN1 protein, the flanking sequences of cTD4 from N- and C-termini form the second anti-parallel β-sheet structure. Therefore, in the context of eTD4, deleting the flanking sequences may disrupt the structure and result in loss of interaction between TDRD1 and Coilin. We further performed Co-IP experiments to confirm this by generating additional mutants on full-length TDRD1(Fig. 6D). As expected, the deletion of C-terminal flanking sequence of eTD4 markedly reduced the interaction between TDRD1 and Coilin, but deletion of N-terminal flanking sequence of eTD4 did not affect the interaction (Fig. 6E). This is a discrepancy in Coilin interaction between deletion mutants of full-length TDRD1 and eTD4. Because TDRD1 has four extended Tudor domains and is structurally highly flexible, it is possible that certain sequences from other eTDs could form the additional anti-parallel β-sheet structure and compensate for the deletion of a.a. 911–990. Collectively, these results suggest that TDRD1 interacts with methylated snRNP proteins through the cTD4 domain in the cytoplasm and can also interact with Coilin through the extended TD4 domain in the nucleus.

Next, we set to delineate the TDRD1-interacting region on Coilin protein. It has been reported that Coilin contains a self-association domain (SA) at the N-terminus, an RG (Arginine-Glycine-rich) box in the center, and a Tudor domain at the C-terminus[30]. We generated Coilin deletion mutants based on these functional domains and tested their interaction with the full-length TDRD1 as well as eTD4 (Fig. 6F). Interestingly, deletion of the RG box completely abrogated the interaction, indicating that the RG box is essential for Coilin to interact with TDRD1 (Fig. 6G and 6H). The RG box contains 33 amino acids harboring 6 GRG tripeptides, which are considered the consensus recognition sequence for PRMT5[31]. Using the PRMT5-specific inhibitor EPZ015666, we observed significantly reduced interaction between TDRD1 and Coilin (Fig. 6I), indicating that the interaction between TDRD1 and Coilin is also PRMT5-dependent.

### Co-expression of TDRD1 and Coilin in human tissues

The physical association between TDRD1 and Coilin proteins strongly argues that these two proteins have a functional link, which prompted us to examine their expression pattern in different human tissues. By searching the RNA expression profiles of Coilin and TDRD1 in the Human Protein Atlas, we found that both genes share very similar tissue expression patterns. They both are highly expressed in testis, but much less in other tissues, in contrast to the broad tissue expression pattern of PRMT5 (**Fig. S4A**). In normal testis samples and prostate tumors, the mRNA levels of TDRD1 and Coilin are positively correlated, with Pearson’s *R* = 0.49 and 0.42, respectively. In contrast, no correlation is observed in normal prostate tissue samples from GTEx or TCGA databases (**Fig. S4B**). We also found that TDRD1 mRNA level positively correlates with PRMT5 and SNRPD3 mRNA levels in prostate tumors, further supporting the functional cooperation of these proteins in prostate cancer (**Fig. S4B**).

### TDRD1 ablation deregulates CB formation and activates p53

**Figure 7:**
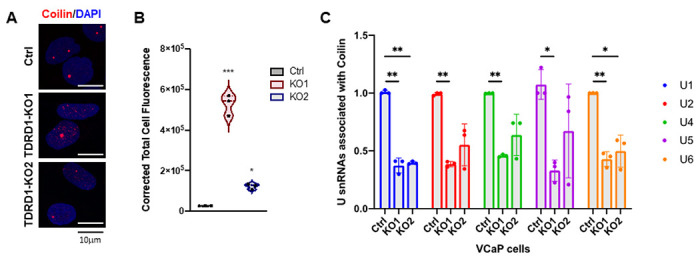
TDRD1 ablation deregulates Cajal body formation and small RNA production. ***(A)***Representative pictures of immunofluorescent staining of endogenous Coilin in VCaP cells. ***(B)*** Quantification of Corrected Total Cell Fluorescence of Coilin in TDRD1 knockout VCaP cells. * p<0.05. ***, p<0.001. ***(C)*** Quantification of major U snRNAs associated with Coilin. Total RNA was recovered from immunoprecipitation of Coilin, and RT-qPCR was performed to quantify the major U snRNAs associated with Coilin. Samples were triplicated. *, p<0.05. **, p<0.01 by Student *t*-test.

The physical interaction between TDRD1 and Coilin suggests that TDRD1 may play a role in the organization of Cajal bodies. Thus, we determined the subcellular localization of Coilin in TDRD1-KO cells. Whereas Coilin proteins usually localize in 1–3 large nuclear bodies in wildtype VCaP cells, they formed multiple nucleoplasmic microfoci when TDRD1 was ablated (Fig. 7A). The overall fluorescence signal of cellular Coilin was increased in both TDRD1-KO lines (Fig. 7B). The alteration of Coilin subcellular localization suggested that snRNP assembly might be affected by TDRD1 deficiency. We then quantified the five major Coilin-associated U snRNA by RNA-immunoprecipitation (RIP). All five snRNAs, including U1, U2, U4, U5, and U6, showed reduced interaction with Coilin in TDRD1 KO cells, indicating that ablation of TDRD1 affects the assembly of snRNP molecules (Fig. 7C).

Similar patterns of microfoci appearance were previously observed when cells were infected with adenovirus or treated with UV or RNA polymerase II inhibitor DRB [32–34]. Cajal Body has been considered a stress-responsive domain, and the microfoci localization of Coilin is often linked to p53 activation. We then examined the levels of p53 total protein and its activated form p53-pSer15. We observed a substantial increase in p53-pSer15 level in TDRD1-KO cells. In line with this, the protein level of p53 target gene p21 was also elevated (**Fig. S5A**). Because p21 is a cyclin-dependent kinase inhibitor and functions as a regulator for G1/S transition, we then examined if the cell cycle was altered in TDRD1-KO cells. As shown in **Fig. S5B**, The percentage of cells in the G1 phase was significantly higher in TDRD1-deficient cells, consistent with the elevated levels of p21. This result further validated the function of TDRD1 on the regulation of cell proliferation.

### TDRD1 regulates the sensitivity of antiandrogens in prostate cancer cells

**Figure 8:**
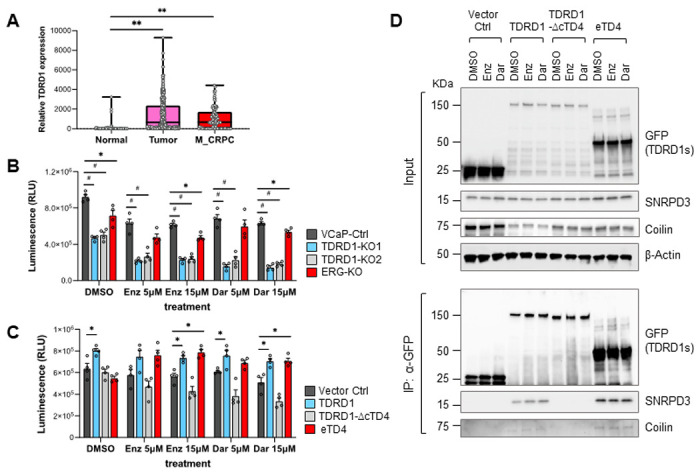
TDRD1 contributes to the resistance of prostate cancer cells against antiandrogens. ***(A)*** TDRD1 gene expression in normal, primary tumor, and metastatic CRPC tissues in TCGA_PRAD and WCDT_MCRPC datasets. Normal, N=52. Tumor, N=500. Metastatic CRPC (M_CRPC), N=99. **, p<0.01 by Student *t*-test. ***(B)*** TDRD1 depletion in VCaP cells increased sensitivity to antiandrogen treatment. Cells were treated with the vehicle DMSO or listed antiandrogens for three days before the cell viability was determined by CellTiter Glo assay. Enz: enzalutamide. Dar: darolutamide. N=4, SEM. *, p<0.05. #, p<0.001. ***(C)*** Exogenous expression of eTD4 enhances antiandrogen resistance in 22Rv1 cells. Cells were transfected with listed plasmid DNAs, followed by antiandrogen treatment 24 hours later. Cell viability was determined after 48 hours of antiandrogen treatment. N=4, SEM. *, p<0.05. (***D***) Co-IP experiment to examine the interaction of TDRD1 mutants with SNRPD3 and Coilin in 22Rv1 cells. Cells were transfected and then treated with 15 uM of antiandrogens for 6 hours before harvesting.

Antiandrogens are often used to treat advanced stages of prostate cancer, especially when cancer has developed castration resistance[35, 36]. Analysis of TDRD1 mRNA expression in primary prostate tumors and metastatic castration-resistant prostate cancer (CRPC) tumors from the TCGA database revealed that TDRD1 expression is maintained in metastatic CRPC tumors, suggesting that TDRD1 may play a role in CRPC cells (Fig. 8A). Given that TDRD1 regulates VCaP cell proliferation, we investigated whether TDRD1 affects cell proliferation under antiandrogen treatment. We selected Enzalutamide and Darolutamide for testing, as these two second-generation antiandrogen drugs possess distinct chemical structures[37]. As shown in Fig. 8B, VCaP cells with TDRD1 deletion, but not ERG deletion, appear to be more sensitive to antiandrogen treatment compared to control knockout cells. To confirm this observation, we transiently expressed the full-length TDRD1, TDRD1 without the cTD4 domain, and eTD4 in 22Rv1 CRPC cells. Full-length TDRD1 or eTD4 expression decreased sensitivity to Enzalutamide and Darolutamide treatment, whereas TDRD1 without cTD4 domain has the opposite effect, indicating TDRD1 regulates antiandrogen sensitivity in 22Rv1 cells (Fig. 8C). Similar experiment was performed in androgen-sensitive LNCaP cells. Although the trend was similar, the impact of TDRD1 overexpression on regulating antiandrogen sensitivity was not as pronounced in 22Rv1 cells. (**Fig. S6**). Therefore we performed a Co-IP experiment in 22Rv1 cells. We found that full-length TDRD1 and eTD4 remain associated with SNRPD3 and Coilin in the presence of antiandrogens, and loss of the cTD4 domain abolished interaction and regulation of antiandrogen sensitivity (Fig. 8D).

## Discussion

In this study, our results revealed an essential role of TDRD1 in regulating the proliferation of TDRD1-positive prostate cancer cells. Ablation of TDRD1 by CRISPR-Cas9 resulted in significantly reduced cell proliferation in both cultured VCaP cells and xenografted tumors grown in mice. We further identified TDRD1-interacting proteins from cytoplasmic and nuclear fractions, and our results showed that TDRD1 is associated with important proteins involved in snRNP assembly in both cellular compartments. Moreover, depletion of TDRD1 in VCaP cells resulted in aberrant subcellular distribution of Coilin, which led to the disorganization of U snRNP complexes and reduced cell proliferation.

Our study identifies a novel PRMT5-TDRD1 signaling axis in the management of cell growth in prostate cancer cells. The involvement of PRMT5 and the high incidence of TDRD1 overexpression in clinical prostate tumor samples strongly indicate that the PRMT5-TDRD1 axis is largely relevant to prostate cancer cell survival, but this mechanism has never been explored before. Previous studies on the role of PRMT5 in prostate cancer have mainly focused on its nuclear functions and its implication in transcriptional regulation. For instance, PRMT5 methylates core histones to regulate the expression of androgen receptor (AR) and its target genes[9, 38]. In ERG-positive cells, PRMT5 methylates AR in an ERG-dependent manner, alters AR chromatin association to genes that regulate prostatic epithelium differentiation, and consequently promotes cell proliferation[39]. In this study, we have demonstrated that the cytoplasmic PRMT5 is also important for prostate cancer cell proliferation in the presence of TDRD1. Cytoplasmic PRMT5 methylates Sm proteins to initiate snRNP complex assembly, and TDRD1 interacts with Sm proteins to facilitate this process in an arginine methylation-dependent manner. These findings are consistent with a previous report that PRMT5 mainly localizes in cell nuclei in the benign prostate epithelium but localizes to the cytoplasm in prostate cancer tissues[11].

Our work suggests an important role of TDRD1 in the regulation of Cajal bodies, which are only observed in nuclei of proliferative cells and metabolically active cells, such as tumor cells, embryonic cells, or neurons. Cajal bodies are membrane-less condensates. Similar to other subcellular structures that are liquid-liquid phase separated, Cajal bodies undergo dynamic changes in response to cellular stress or signals[40–42]. It was previously reported that the composition and substructure of Cajal bodies are defined by specific interaction between dimethylarginines and Tudor domain-containing proteins[43]. Similarly, TDRD1 forms condensates both in the cytoplasm and in the nucleus, and its eTD4 domain is responsible for appropriate cellular localization. At the molecular level, we found that eTD4 interacts with both Sm proteins and Coilin in an arginine dimethylation-dependent manner. Without TDRD1, Cajal bodies changed their morphology. All these observations are in agreement with the concept that dimethylarginine-Tudor interaction modules contribute to the dynamics of cellular condensates[43].

As an epigenetic enzyme that modifies both core histones and non-histone substrates, PRMT5 is an emerging cancer therapeutic target and its specific inhibitors have been developed and tested in many preclinical studies[44, 45]. Recently several clinical trials using PRMT5-selective inhibitors were initiated for the treatment of advanced or metastatic tumors. However, PRMT5 is broadly expressed in all major tissues in mammals and regulates multiple biological pathways, including but not limited to RNA processing, metabolism, and splicing[46]. PRMT5 knockout mice were embryonic lethal, indicating that PRMT5 is an essential gene for embryonic development[47]. Our study indicates that TDRD1 is an alternative therapeutic target in the PRMT5-TDRD1 axis. Under normal physical conditions, TDRD1 is only expressed in germ cells in men, but not in other types of cells/tissue (**Fig. S3A**). This tissue-specific expression of TDRD1 indicates that targeting TDRD1 may have fewer side effects, in contrast to targeting PRMT5. Consistently, complete knockout of TDRD1 in mice did not develop any observed abnormality except for the defect of spermatogenesis in male mice, indicating that TDRD1 is a non-essential gene for development and survival[48].

One limitation of this study is that we could only conduct the gene depletion experiments on one single prostate cancer cell line, VCaP, the only TDRD1-positive prostate cancer cell line widely used in the field. Nevertheless, TDRD1 mRNA and protein are overexpressed in more than half of the prostate tumors in human patients, including CRPC tumors, supporting its importance in prostate cancer development[1]. Furthermore, the overexpression experiments in 22Rv1 cells confirmed TDRD1 ’s association with snRNP machinery proteins and reinforced its role in cell proliferation during antiandrogen treatment. When more TDRD1-positive cell lines and PDX models are available, these tools will improve our understanding of TDRD1 function and offer a potential new target for treating prostate cancer patients.

## Data Availability

All data generated or analyzed during this study are included in this published article, supplementary information slides, or available from the corresponding authors on reasonable request.
